# Spinal Vascular Shunts: Single-Center Series and Review of the Literature of Their Classification

**DOI:** 10.3390/neurolint14030047

**Published:** 2022-07-15

**Authors:** Jafeth Lizana, Nelida Aliaga, Walter Marani, Amanda Escribano, Nicola Montemurro

**Affiliations:** 1 Department of Neurosurgery, Hospital Nacional Guillermo Almenara Irigoyen, Lima 07035, Peru; jafethlizana@gmail.com; 2 Department of Medicine, School of Biomedical Sciences, Austral University, Buenos Aires B1751, Argentina; nelida.aliaga.s@gmail.com (N.A.); amandaescriar@gmail.com (A.E.); 3 Division of Neurosurgery, Department of Basic Medical Sciences, Neurosciences and Sense Organs, University “Aldo Moro” of Bari, 70100 Bari, Italy; wmarani1@gmail.com; 4 Department of Neurosurgery, Azienda Ospedaliera Universitaria Pisana (AOUP), University of Pisa, 56100 Pisa, Italy

**Keywords:** spinal vascular malformations, spinal arteriovenous fistulas, spinal arteriovenous malformations, sAVF, sAVM, neurosurgery, embolization, clinical outcomes

## Abstract

Spinal arteriovenous shunts (sAVSs) are an uncommon disease, constituting 3 to 4% of intradural lesions; 70% of these lesions are spinal arteriovenous fistulas (sAVFs), whereas spinal arteriovenous malformations (sAVMs) are rarer. Both share the problem of their classification due to the heterogeneity of their angioarchitecture. The aim of this study is to report a series of sAVSs treated in the neurosurgery department of the Hospital Nacional Guillermo Almenara during the 2018–2020 period and to present an overview of the current literature on sAVS classification. We reviewed all medical records of patients diagnosed with sAVFs and sAVMs during the 2018–2020 period, and then we analyzed images with Horos v4.0.0, illustrated some cases with Clip Studio Paint v1.10.5, and performed a descriptive statistical analysis with SPSS v25. Twelve patients were included in this study, eight of which were women (67%) and four of which were men (33%); the age range was from 3 to 74 years. Eight sAVSs were sAVFs, whereas four were sAVMs. The most frequent clinical manifestation was chronic myelopathy in seven patients (58%). Of those treated only by embolization, seven (70%) resulted in complete occlusion (five sAVFs and two sAVMs), while three (30%) remained with a residual lesion. At last follow-up, five patients (42%) improved clinically, and the seven remaining (58%) maintained the same neurological state. sAVSs require a detailed study of their angioarchitecture for proper management. The endovascular treatment is safe with acceptable cure rates. The surgical option should not be set aside.

## 1. Introduction

Spinal arteriovenous shunts (sAVSs) are a group of uncommon and heterogeneous diseases, constituting 3–4% of intradural lesions as well as 5–9% of the vascular pathologies of the central nervous system [1,2,3]. The annual incidence of spinal arteriovenous fistulas (sAVFs) is 5 to 10 per million. Spinal dural arteriovenous fistulas (sDAVFs) are the most frequent type (60 to 80% of the total); they have a predilection for the male sex (5:1); they are more frequent in older adults, as two-thirds are diagnosed around the sixth or seventh decade of life; and 90% of sDAVFs are located between levels T4 and L3 [4,5,6,7,8]. Intradural sAVFs are less frequent, showing no predilection for any gender, and occur at younger ages [7,8,9].

Spinal arteriovenous malformations (sAVMs) usually appear in the third decade of life, and it is not uncommon to find them in pediatric age, in some cases associated with genetic syndromes. sAVMs can cause acute, subacute, or chronic spinal cord dysfunction [8]. Most of them are located at the thoracic or lumbar level (70%), and there is no gender predilection [10,11,12]. These patients commonly present for neurosurgical attention after a protracted course with severe neurological dysfunction. They share some characteristics with their intracranial counterparts, but their clinical impact is often comparatively worse [8]. For this reason, an early and correct recognition of the pathology is mandatory to halt the progression of the disease and minimize permanent spinal cord injury.

Regarding the AVFs of the cranio-cervical junction (CCJ AVFs), they are a group of complex and infrequent lesions (1 to 2% of the AVFs of the CNS); they are defined as an arteriovenous shunt at the epidural, dural, or intradural level, located between the foramen magnum and C2 [13,14]. The aim of this paper is to show our single-center experience with pathology and to perform a detailed review of classification, anatomical characteristics, and management implications.

Furthermore, it should be noted that these lesions are biologically active and thus are prone to angiogenesis, vascular remodeling, and thrombosis [15]. Although in most cases the injuries are unique, patients with certain genetic pathologies often develop multiple injuries, both spinal and extra-spinal [16,17]. In these genetic cases, we differentiate hereditary and non-hereditary [17]. The first refers to pathologies such as hereditary hemorrhagic telangiectasia (HHT) and RASA1 gene mutations [17,18]. The second is commonly called metameric (Cobb syndrome); however, it can occur as part of syndromes such as Klippel–Trenaunay or Parkes Weber [15,17,18]. In the case of hereditary lesions, mutations in the endoglin and transforming growth factor-β genes affect vascular cells in their germinal stage [17,18]. While metameric lesions have their origin in later stages of embryogenesis, it should be considered that spinal cord endothelial cells share the same mesodermal origin as skin and muscle [17].

## 2. Materials and Methods

We performed a retrospective review of clinical records and medical images of patients with sAVSs. To achieve the purpose of this paper, we defined sAVSs as a term that encompasses sAVMs and sAVFs. We included cases treated between January 2018 and December 2020, with a follow-up of at least 1 year. We described their clinical characteristics, angioarchitecture, classification, procedures for management, and outcomes. We used the Modified Rankin Scale to quantify the outcome of our patients. The reconstruction and analyses of clinical images were performed with Horos v4.0.0. Illustrations of the cases and images were made using Clip Studio Paint v1.10.5. We identified general demographic, clinical, and imaging characteristics from the database of patients operated on by neurosurgeons at our institution. No patients were excluded during this period. In our center, endovascular treatment is the first line of management for these pathologies performed by a single neuroradiologist, and therefore we focused on endovascular management, the number of procedures performed, and their outcomes. However, surgical management was considered when there was evidence of spinal cord compression, when the lesion was not susceptible to the endovascular approach, or when embolization failed. We used the Modified Rankin Scale to describe and analyze the functional status of the patients at admission time and at last follow-up. All patients underwent preoperative and postoperative spinal angiography. However, not all patients had spinal cord MRI or angio-CT. In this retrospective single-center study, descriptive statistical analyses were used to collect data, and data were combined into tables for comparison. The limited data availability was not sufficient to perform specific statistical analysis. Furthermore, a PubMed, MEDLINE, and Scopus review was carried out to identify studies dealing with this pathology. In the present study, we will preferably use the Spetzler classification; however, this does not include many of the injuries described, and for that reason we will use other classifications when required.

## 3. Results

### 3.1. Clinical Series

Twelve consecutive patients with sAVSs were treated between January 2018 and December 2020. Of the 12 patients, 8 were women (67%) and 4 were men (33%) (Table 1). The age range was from 3 to 74 years, with a mean of 28 years old. In the case of the sAVMs, the mean was 37 years, and for the sAVFs, the mean age was 24 years.

Most of our cases were located at the thoracic level, with six patients (50%); two were at the cranio-cervical junction (17%), and four cases (33%) were at the CM. Regarding the number of afferents, nine cases (75%) of pial afferents were found, two patients (17%) had both dural and pial afferents, and one patient (8%) had only dural afferents. Chronic myelopathy occurred in seven patients (58%), acute myelopathy occurred in three cases (25%), and two patients (17%) presented SAH. Flow aneurysms were developed by six patients. All clinical management and outcome characteristics are shown in Table 2.

### 3.2. AVMs

Regarding the angioarchitecture, 50% of the sAVM cases had a compact nidus and 50% had a diffuse nidus. Furthermore, the most frequent location was in the conus medullaris (CM) (three patients). Concerning the number of afferents, 100% of the sAVMs had pial afferents, and the average number of afferents was two per AVM case. The venous drainage of the sAVMs was 100% intradural. Moreover, flow aneurysms occurred in one patient. This was an arterial and venous aneurysm, in a CM AVM; however, it had an important fistulous component.

### 3.3. AVFs

The most frequent location was at the lower thoracic levels. It was observed that the radiculomedullary artery (RMA) was one of the afferents in three patients (37.2%), two patients (25%) had the radiculomeningeal artery (RMEA) as an afferent, and two patients (25%) had the posterior spinal artery (PSA). Meningeal afferents were present in the epidural AVF and CCJ AVF cases. Regarding the venous drainage of the sAVF, four patients (33%) had extradural drainage, corresponding to epidural AVF, CCJ AVF, ventral AVF, and CM AVF. Additionally, five patients (62.5%) developed flow aneurysms, two arterial and three venous aneurysms from sAVF and CCJ AVF cases. Venous aneurysms were defined as an intradural saccular image in the spinal digital subtraction angiography (sDSA) with a diameter greater than a threshold.

### 3.4. Management and Outcome

The management was predominantly endovascular (10 cases); one patient underwent combined treatment, and one case had spontaneous thrombosis. The patient with combined management resulted in total occlusion. Occlusion was total in 75% of patients with sAVMs and in 71% of patients with sAVFs (Table 3).

The first control with sDSA occurred between 6 months and a year after embolization. Of the patients submitted to embolization alone, seven patients (70%) resulted in total occlusion, while in three patients (30%) there was residual lesion (Table 3). Histoacryl was used in all patients, and in one case it was associated with platinum coils. None of the cases of partial embolization had rupture in the follow-up. Regarding the outcome, we had 10 patients with an mRS of 3 or 4 at admission; only two patients had an mRS less than 3 at admission. In the follow-up, for a period of 1 year to 5 years, five patients (42%) improved clinically. Of these, two patients were left with mild sequelae and three without neurological symptoms. The seven remaining patients maintained the same neurological state. In the case of sAVFs treated, three patients showed improvement in their symptoms (43%), while two patients with sAVMs improved at follow-up.

### 3.5. Clinical Cases

#### 3.5.1. Case 4

**Figure 1:**
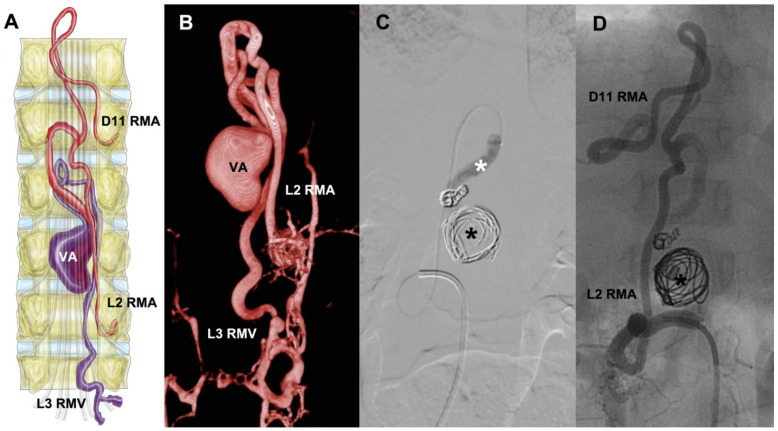
Depiction of a ventral sAVF at medullary cone schematized (**A**). It has two feeders at right D11 and L2 RMA, and then it shows a venous aneurysm followed by its drainage through L3 RMV. (**B**) The 3D sDSA reconstruction, followed by the embolization process and the exclusion of the lesion (**C**,**D**). RMA = radiculomedullary artery, RMV = radiculomedullary vein, VA = venous aneurysm, black asterisk = coils inside VA, white asterisk = Histoacryl at the sAVF. Illustrated by J. Lizana.

A 3-year-old female with developmental delay and lumbar pain had undergone L1 laminectomy when she was 1 year old. At admission, she had paraparesis 4/5. sDSA showed a lesion with fistulous ostium at D12-L1, a venous aneurysm, afferents from right D11 and L2 radiculomedullary arteries, and venous drainage through the L3 radiculomedullary vein (Figure 1). The procedure was performed with a Rebar 18 microcatheter assisted with a Silver Speed 16 micro-guidewire. We achieved the catheterization of the sAVF. Then, we proceeded to embolization with coils, first MicroPlex 18 Cosmos (14 mm × 51 mm), then six HydroCoils, and finally we instilled Histoacryl. The angiographic control showed total occlusion of the vascular lesion.

#### 3.5.2. Case 7

**Figure 2:**
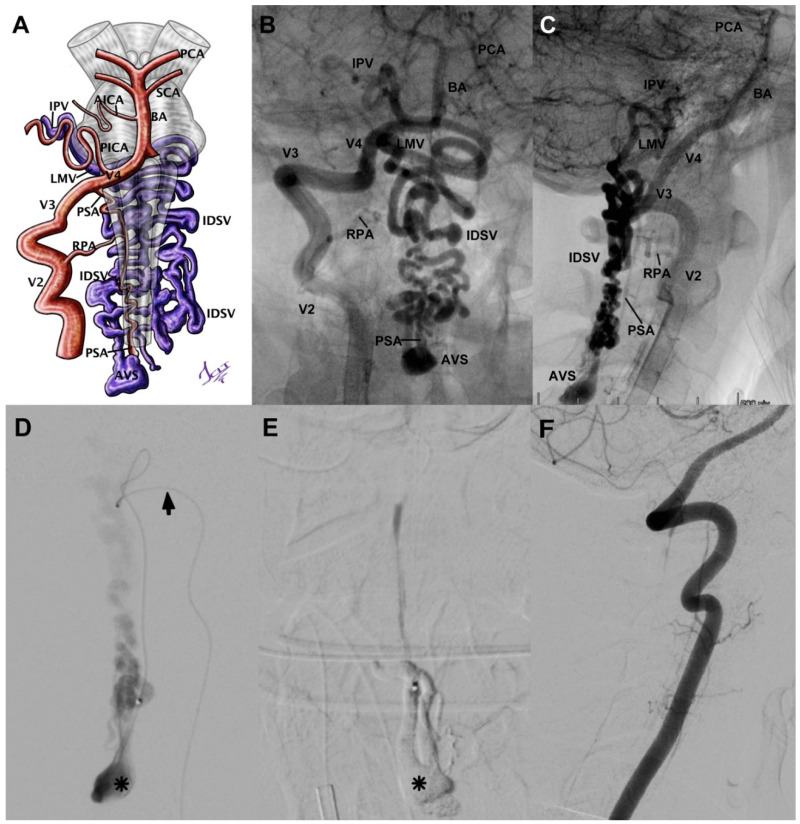
(**A**) Schematic of an AFV case corresponding to the angiographies displayed in images (**B**) and (**C**). We consider a CCJ injury even though the ostium is at a C3 level, due to afferents and intracranial drainage. It shows (**D**) the catheterization through the posterior spinal artery (black arrow) and the venous aneurysm at the ostium level (asterisk). Histoacryl at the level of the fistulous ostium (**E**) and the aneurysmal dilation (asterisk). Angiographic control (**F**) shows complete absence of the lesion. BA = basilar artery, SCA = superior cerebellar artery, AICA = anteroinferior cerebellar artery, PICA = posteroinferior cerebellar artery, PCA = posterior cerebral artery, V2 = foraminal segment of vertebral artery, V3 = atlantic segment of vertebral artery, V4 = intradural segment of vertebral artery, PSA = posterior spinal artery, RPA = radiculopial artery, AVS = arteriovenous shunt, IDSV = intradural spinal vein, LMV = lateral medullary vein, IPV = inferior petrosal vein. Illustrated by J. Lizana.

Case 7 was a 15-year-old female with SAH Fisher IV and negative angio-CT. An sDSA was performed, and it showed a CCJ AVF with afferent from PSA, the fistulous ostium also located at C2-C3, a venous aneurysm in contiguity, and the venous drainage toward the intradural varicose vein (Figure 2A–C). Subsequently, we proceeded to micro-catheterize the fistulous ostium and embolize it with Histoacryl (Figure 2D), achieving its total occlusion (Figure 2E,F). The patient was released without neurological sequel.

#### 3.5.3. Case 8

**Figure 3:**
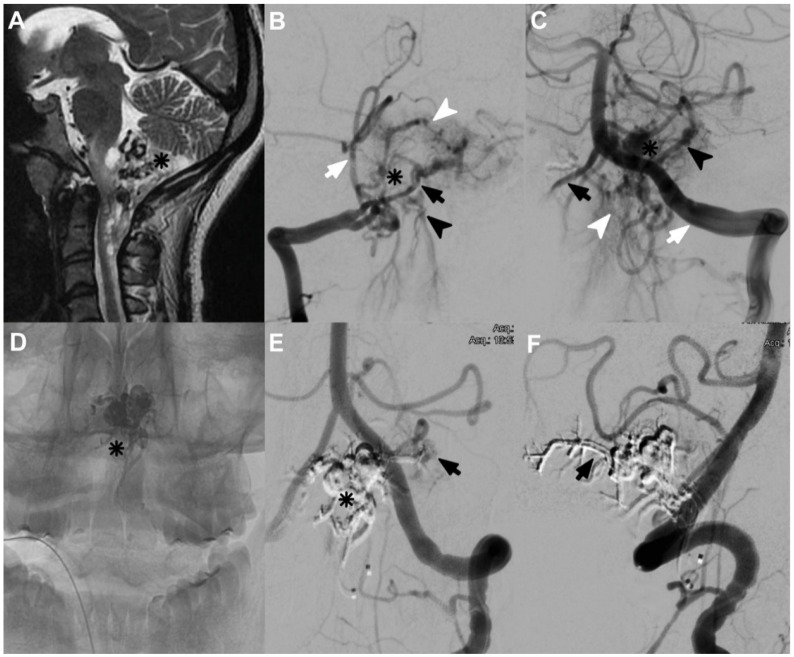
(**A**) The T2 MRI of a complex AVF of the CCJ (asterisk). (**B**) The AP DSA of the right vertebral artery (VA); an extracranial PICA is noted (white arrow), as is a small arterial aneurysm (asterisk). Hypoplasia of the V4 segment (black arrow) and an arterial shunt (white arrowhead) are also evident. Venous drainage towards the marginal sinus and the extradural venous plexus (black arrowhead) can also be observed. (**C**) The AP left VA (white arrow); the hypoplastic right VA is noted (black arrow). Furthermore, the intradural PICA (black arrowhead), the dysplastic aneurysm (asterisk), and the extradural venous drainage (white arrowhead) can be observed. (**D**) Histoacryl on fluoroscopy. (**E**) The obstructed CCJ AVF, including the aneurysm (asterisk), is shown, but a small residual component can be noted (black arrow). (**F**) The left VA, showing the patency of the PICA, but a complete occlusion of the posterior meningeal artery (black arrow).

Case 8 was a 26-year-old female with a history of two syncopal episodes. The MRI showed void signals at the CCJ. Angiography resulted in CCJ AVF (Figure 3A), with feeders from PSA, which arose from extracranial PICA on the right side and RMA with posterior meningeal branches from V3 on the left side. We identified two arterial aneurysms, and the drainage was toward marginal sinus and subdural veins (Figure 3B,C). In the first session, we embolized with Histoacryl through the right PSA (Figure 3D). The second session was through a left RMA. Angiographic control one year later showed a very small remnant of CCJ AVF (Figure 3E,F), but the patient did not develop any neurological deficit.

#### 3.5.4. Case 10

**Figure 4:**
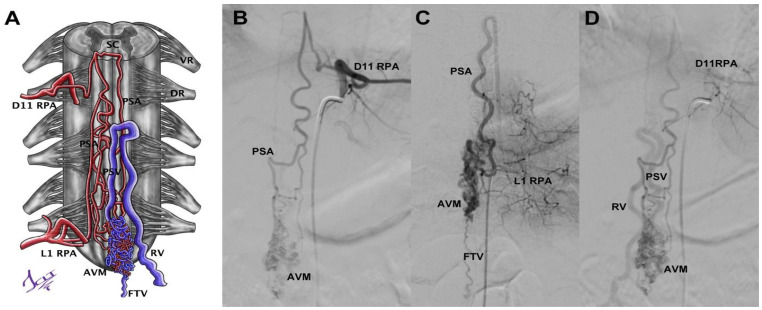
A compact AVM of the medullary cone schematized (**A**) from the angiographies through the left intercostal artery at the D11 level (**B**), the left lumbar artery at the L1 level (**C**), and their venous phase (**D**). RPA = radiculopial artery, AVM = AVM nidus, PSA = posterior spinal artery, DR = dorsal root, VR = ventral root, PSV = posterior spinal vein, RV = radicular vein, SC = spinal cord, FTV = filum terminale vein. Illustrated by J. Lizana.

Case 10 was an 18-year-old male with sudden lumbar pain, paraparesis, headache, and nausea. Brain CT showed subarachnoid hemorrhage (SAH), and sDSA displayed a vascular lesion. The nidus was located from D12 to L2, and the afferents proceeded from left D11 and left L1 (radiculopial arteries (RPAs) and PSA); its drainage was through the radiculomedullary vein (RMV) (Figure 4). However, the Wada test performed in L1 was positive, and we decided to embolize the left D11 afferent with Histoacryl. Four months later, a new Wada test through left L1 was negative, so we proceeded to embolize with Histoacryl, achieving total occlusion of the sAVM without injury.

#### 3.5.5. Case 11

**Figure 5:**
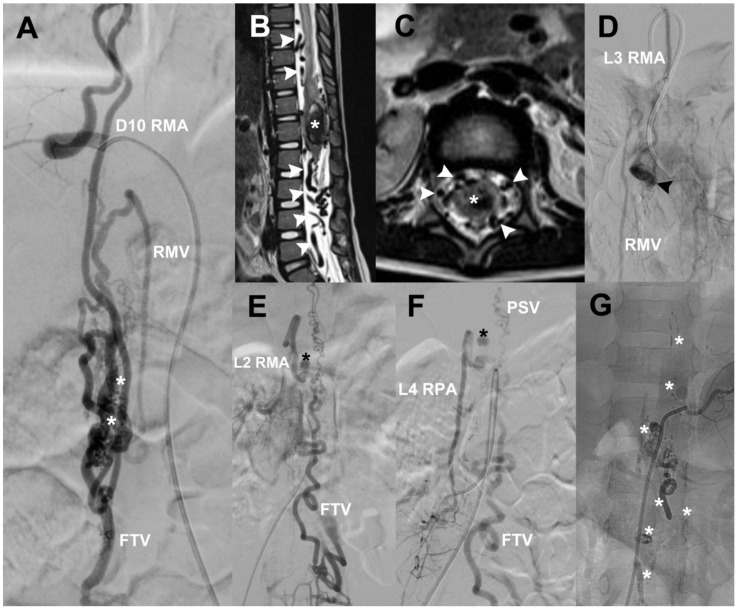
(**A**) A diffuse CM sAVM case; white asterisks mark the place of the diffuse nidus, and the first afferent is right D12 RMA while the drainage goes to RMV and FTV. Lumbosacral T2-MRI shows flow voids (white arrowheads) and hematomyelia (white asterisks) (**B**,**C**). (**D**) One fistulous component at left L3 RMA; a venous aneurysm (black arrowhead) and its drainage through RMV. Other fistulas are seen at right L2 RMA (**E**) and right L4 RPA (**F**) while their drainage goes through PSV and FTV; a small arterial aneurysm (black asterisk) can be seen. (**G**) A negative control sDSA after Histoacryl embolization (white asterisks) and surgical management. CM = conus medullaris, RMA = radiculomedullary artery, RPA = radiculopial artery, RMV = radiculomedullary vein, FTV = filum terminale vein, PSV = posterior spinal vein.

A 5-year-old male with suspicion of Cobb syndrome developed sudden paraplegia, pain in both legs, and sphincter incontinence. MRI showed void signals around the CM and hematomyelia. sDSA was made and showed a diffuse and fistulous sAVM at CM, with afferents from right D10, L2 RMA, left L3 RMA, and L4 RPA with a diffuse nidus at L1–L3, and venous drainage towards RMV, FTV, and PSV. We found an arterial aneurysm at L2 and a venous aneurysm at L3 (Figure 5). First, the right D10 artery was catheterized and then embolized with Histoacryl, resulting in complete occlusion of the nidus. Because of hematomyelia, the patient was operated on for partial resection of the sAVM, evacuation of hematoma, and laminoplasty. Seven months later, he was submitted to embolization through left L3 RMA, right L2 RMA, and right L4 RPA. After the procedure, we achieved complete occlusion of the lesion; at that time, the patient’s neurological status improved to paraparesis 4/5. The 6-month and 1-year control sDSA did not show any vascular lesions.

## 4. Discussion

### 4.1. Pathophysiology and Clinics

Most sAVFs have a low risk of rupture, their symptoms are related to venous congestion and secondary compression, which is explained by the presence of valves between the fourth and third orders of the medullary veins that prevent intramedullary reflux [19]. Its paucisymptomatic and chronic course generates a delay in diagnosis. The most common symptoms are lower limb weakness, paresthesia, low back pain, autonomic dysfunction, and, more rarely, Foix Alajouanine syndrome (subacute necrotizing angiodysgenic myelopathy) [12,19,20]. In the case of intradural sAVMs, 50% present with rupture. Glomus-type sAVMs have a risk of rupture like brain AVMs (4% per year). However, their rupture increases the rebleeding risk to 10% during the first month and to 40% in the first year, and then it progressively decreases during the following 10 years. The risk of bleeding may be due to the association with arterial aneurysms (up to 29% of cases). The mortality of each bleeding episode ranges from 10 to 20% [11,21,22,23].

The presentation of CCJ AFV is varied (SAH, myelopathy, brainstem syndromes, and cranial nerve deficit). The risk of rupture is the highest (37%), and the risk of rebleeding can be as high as 60%. In 10% of the cases, it is associated with flow aneurysms proximal to the lesion, and 10% of patients debut with brainstem or spinal cord hematoma [13,14].

### 4.2. Classification

The classification of sAVMs and sAVFs is a subject of discussion and controversy. In 1971, Di Chiro classified sAVFs into three categories: type I (single screwed vessel), type II (glomus), and type III (juvenile) [24]. Later, Djindjian in 1977 and Hero in 1986 reported ventral spinal shunts dependent on the ASA, and in 1987, Rosenblum named them as direct sAVFs or type IV [9,25]. Then, in 1993, Mourier proposed subclassifying them by the size of the ostium and the number/caliber of the afferents: subtypes I, II, and III [26]. Later, these subtypes were named A, B, and C, respectively. Mourier concluded that subtype III (subtype C) is susceptible to embolization. On the contrary, subtype I (subtype A) is susceptible to surgery, and in subtype II (subtype B), both routes are effective (Table 4) [24,26,27].

In 2002, Spetzler classified spinal shunts from a surgical point of view, separating AVFs from AVMs. sAVFs are classified as epidural, intradural dorsal (Di Chiro type I), and intradural ventral (Rosenblum type IV) [24,27,28]. In 2011, Rangel-Castilla subclassified epidural (extradural) AVFs into three types: type A (intradural venous drainage), type B1 (without intradural venous drainage and with neurological deficit), and type B2 (without intradural venous drainage and without neurological deficit) [24,28,29]. In 2002, Rodesch also proposed a classification based on the pathophysiology of vascular lesions and their genetic background [17]. In 2017, Takai reviewed the standard classifications and added type V, which he defined as an extradural AVF and in turn subclassified it into type A (with spinal drainage) and type B (without spinal drainage) [30]. In 2018, Adeeb classified dorsal sAVFs, adding the subtype A for those with dorsal venous drainage and subtype B for those with ventral venous drainage [31]. Spetzler and Kim organized sAVMs into three types: extra- and intradural AVMs (metameric, juvenile, or Di Chiro type III), intramedullary sAVMs (including Di Chiro type II), and CM AVMs, the latter having unique characteristics (arterial anastomosis, arterial afferents, multiple glomus nidus, multiple shunts, and complex venous drainage). Two subtypes are distinguished for intramedullary sAVMs: the compact lesion (glomus) and the other diffuse lesions (Table 4) [12,24,27,28,32].

Another classification, proposed by Zozulya in 2006, tried to unify aspects of the previous classifications, adding intravertebral malformations [33]. This classification describes anatomical, hemodynamic, and angioarchitectural characteristics [33]. Nevertheless, the complexity of the proposal makes its use very difficult in practice. Finally, the above classifications do not consider paraspinal AVFs and filum terminale AVFs, both of which have unique clinical, radiological, and anatomical characteristics [34,35,36,37].

The CCJ AVFs cannot be included in the previous classifications. In 2008, Geibprasert classified epidural CCJ AVFs embryologically, resulting in three types: ventral, dorsal, and lateral, the latter being the one with the highest risk of rupture (Table 5) [38]. In 2017, Hiramatsu detailed the angioarchitecture of 54 patients with CCJ AVFs and defined five types: type I (dural AVF), type II (radicular AVF), type III (epidural AVF with pial afferents), type IV (epidural AVF without pial afferents), and type V (pial AVF) (Table 6) [13]. In addition, it is thought that when the shunt is at the C1–C2 level, its afferents are radicular or radiculomeningeal arteries, and when it is in the foramen magnum (C0), its afferents usually come from the vertebral, occipital, or the ascending pharyngeal artery [13,14]. In Table 1, it can be noticed that some of our cases fit partially or do not fit at all within the classifications shown in Table 4, Table 5 and Table 6.

**Figure 6:**
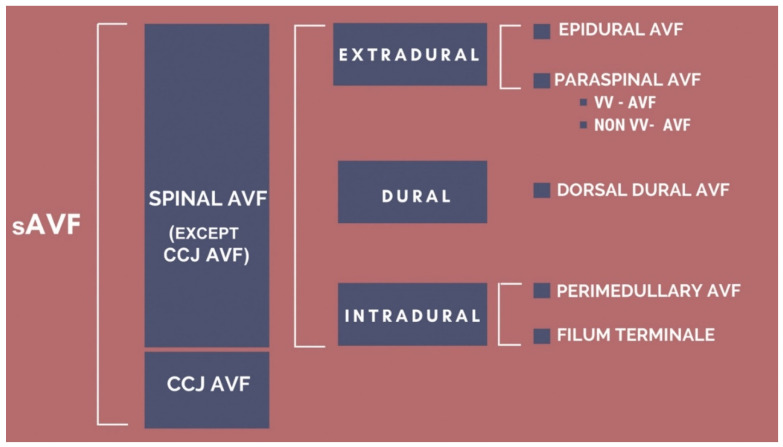
Depiction of how previous sAVF classifications could converge. VV-AVF = vertebro-vertebral AVF, NON VV-AVF = non-vertebro-vertebral AVF.

**Figure 7:**
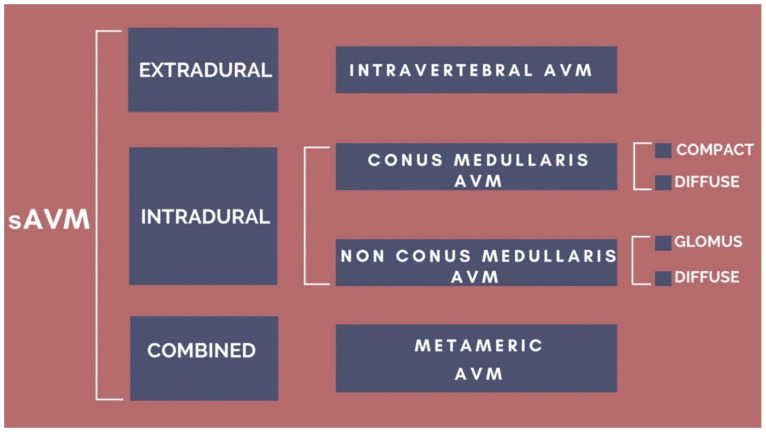
Depiction of how previous sAVM classifications could converge.

In addition, there are similar cases reported in literature, and it continues to be challenging to develop a general classification of this pathology. There will be as many varieties of arteriovenous shunts as the possible anatomical variations allow, so to date there is no classification that satisfies all cases and some cases do not fit in any classification. Yet, when comparing them, we notice that they are complementary to each other in various aspects, as we exemplify in Figure 6 and Figure 7.

In the reported cases, a significant number of patients were pediatric (25%) and female (66.6%). Most of the vascular lesions were CM AVMs, ventral pial AVFs, and CCJ AVFs, hence the high frequency of flow aneurysms. Furthermore, it is important to note that the three cases that presented hemorrhage were CM AVM, CCJ AVF, and ventral pial AVF. With reference to the CM AVM cases, we had patients with diffuse nidus, and due to their implication in management, we propose subclassifying the compact nidus as subtype A and the diffuse nidus as subtype B [28,39]. A case of ventral spinal sAVF, at the D11 level, was found incidentally in a patient with a presumptive diagnosis of a hypervascularized spinal tumor; embolization was planned, and when attempting to perform it, the vascular lesion had disappeared spontaneously.

### 4.3. Management

The surgical management of sAVSs is considered by many centers as the gold standard treatment due to its high rate of effectiveness (between 83% and 98% cure) [40,41,42,43]. Open microsurgery is especially necessary if a vascular formation has the kind of structure in which there is a collateral blood flow and embolization of the main tributaries will not result in complete occlusion of the blood flow, if selective spinal angiography does not identify all tributaries or there are suspiciously few of them, and, of course, if embolization failed [33,44]. Both treatments (microsurgical and endovascular interventions) differentiate according to the type of sAVS [33]. sAVMs and sAVFs of the anterior face constitute a challenge due to the complexity of the approach and the limitation of space; in those cases, the endovascular route is usually more accessible [44,45,46]. In anterolateral lesions, the posterior approach may be made feasible by associating the arachnoid dissection with the dentate ligament section, and even some dorsal root to allow the spinal rotation (in upper cervical and mid-lower thoracic levels without significant deficit) [43,45].

The surgical indication for sAVMs is suggested for compact ones; the pial dissection of the afferents avoids the realization of myelotomy. In this work, it is essential to differentiate the passage vessels from the anomalous vessels, the latter being larger and more tortuous [39,42,47,48]. In the spinal cord, partial resection of an sAVM does not increase the risk of bleeding; most of them evolve to the thrombosis of the residual nidus, maintaining the functionality of the patient [30,38]. In the sAVMs at the CM or the filum terminale, the afferents are usually small, multiple, and difficult to catheterize. There are also communications between the ASA and PSA; in those cases, surgical management is recommended over embolization [22,39,40,49,50,51].

Transient clipping of the drainage vein and indocyanine green video angiography aid in the identification of the shunt. Physiological neuromonitoring is recommended when very eloquent arteries are involved. It is important to keep MAP above 85 mmHg for adequate spinal cord perfusion [41,43,52]. Other important aspects are the hermetic closure of the meninges to avoid CSF fistulas, the possibility of laminoplasty, and the need for instrumentation. The latter will depend on the instability established by the surgery [39,42,53]. Multiple laminectomies (three or more) at the cervical or dorsal level are related to instability, especially in those under 25 years [53].

Intraoperative sDSA is irreplaceable, but femoral access is difficult due to the position of the patient. A protocol for femoral access is reported in said cases, and another option is the use of the radial approach, which opens the possibility of a hybrid treatment of this pathology [54,55]. In the absence of sDSA, the use of indocyanine green video angiography should be considered to assess the degree of resection [39,42,56]. Even though endovascular management has shown effectiveness in most sAVS types, it has been reported to be less effective than surgery, and in many cases, there is a need for more than one session. The success of this approach has improved in recent years, with complete occlusion rates between 70 and 90% [40,41,57,58]. This is due to the advances in devices and materials, which is why in some centers the endovascular route constitutes the primary therapy in said cases [57,59,60]. However, the literature describes that some subtypes (sDAVFs, CM AVMs, and metameric lesions) are less susceptible to endovascular treatment [15,16].

In diffuse and anterior sAVMs, embolization is recommended, but when the afferent arteries are very thin and/or multiple, its feasibility should be evaluated. This scenario constitutes a challenge, and the transvenous approach or surgery should be considered [49,58,59]. The objective of embolization is to close the arteriovenous shunt (or the nidus in the case of sAVMs) [50,59,61] and make the embolizing substance enter the draining vein, while being careful with the reflux towards the afferents [59,62,63].

In cases of CCJ AVFs and CM AVFs, the presence of arterio-arterial shunts is not uncommon; sometimes these shunts compromise eloquent arteries, and this constitutes a risk during embolization [64]. In a recent multicenter study in patients with CCJ AVFs, embolization was found to be the main risk factor for ischemic complications [64]. In this cohort, surgical and endovascular approaches were compared, and the results showed a clear advantage for surgical treatment, both in the success rate and in the number of complications [65]. Similarly, when the endovascular treatments of dural AVFs of the foramen magnum (FM AFVs) and CCJ AVFs were compared, the latter had a lower rate of occlusion and more ischemic complications [66,67]. The embolizing substance used in all our patients was Histoacryl. In one patient, we used coils in a very large ostium with a high risk of embolizer migration; the use of removable catheters to prevent reflux or balloon catheters that can help when there are arterio-arterial communications cannot be ignored [47]. The total embolization rate in this series (70%) is acceptable and is in accordance with the literature. However, it must be considered that many of these injuries were very complex. Furthermore, in the cases where the embolization was partial, the residual lesion was very small, and in the follow-up of 1 to 5 years, none bled or worsened [40,41]. In such cases with partial occlusion, it is important to treat the flow aneurysms first [68]. While embolization is a safe option, we cannot ignore surgery. As the natural course related to pathophysiological events occurs as a result of two subsequent phases such as primary and secondary (delayed) injuries of the spinal cord [69], we showed that one of the most complex cases underwent a combined treatment, with complete occlusion of a lesion, showing that both techniques are not exclusive and together can achieve better results. In the near future, telemedicine and virtual embolizations could support neuroradiologists in treatment planning and in education in clinical practice, supporting a better understanding of these AVSs [70,71,72]. The visual exploration application can be extended by quantitative blood flow information listing pressure drops and further hemodynamic parameters [72].

### 4.4. Limitations of the Study

The main limitations of this study are the small sample of patients and the heterogeneity of these sAVSs due to the existence of a wide range of different types and subtypes of lesions in our small sample. These limitations make it impossible for us to perform a deep statistical analysis or a proper association. All patients were treated with the endovascular procedure, whereas only one patient was treated in a hybrid way. At present, several hospitals treat these lesions with endovascular procedures, while in many cases, surgery could be considered as the gold standard. We believe that surgery or hybrid management must be kept as first-line treatments along with endovascular procedures, and we must keep in mind that one option does not exclude the other ones.

## 5. Conclusions

sAVSs constitute a wide group of rare and heterogeneous pathologies where more studies and consensus are needed. An understanding of their angioarchitecture and hemodynamics is essential for planning surgical or endovascular management. In our institution, the primary management of sAVSs is endovascular, with no relevant complications occurring in the presented cases. Surgical management should not be left aside and should be considered a possibility in all patients, if the patient’s clinical condition allows it, especially when the location or angioarchitecture makes treatment difficult or an embolization attempt failed. The management of each patient must be highly individualized according to our single-center experience.

## Figures and Tables

**Table 1:** Demographic characteristics.

	Spinal AVFs			Spinal AVMs		
Patients	Epidural AVF	CCJ AFV	Pial Ventral AVF	CM AVM	Other AVM	Total
Age <18	-	1	2	1	-	4
Age 18–59	3	1	1	1	1	7
>60	-	-	-	1	-	1
Total	3	2	3	3	1	12

**Table 2:** Clinical management and outcome characteristics of study population.

	Age/Sex	Symptoms	Admission mRS	Classification	Afferents	Ostium/Nidus	Aneurysms	Venous Drainage	Management	Outcome	Last Follow-Up mRS
Case 1	44/F	Chronic myelopathy (paraparesis 2/5, paresthesia, sphincter incontinence)	4	AVF extradural epidural lateral subtype B1 (Rangel-Castilla)	Right L1 radiculomeningeal artery	Small ostium D12	No	Ventral epidural venous plexus	Embolized with Histoacryl	Complete occlusion. Without improvement of the sequel	4
Case 2	18/F	Chronic myelopathy, left leg monoparesis 2/5	3	AVF extradural epidural dorsal sub type A (Rangel-Castilla)	Right radiculopial L1 and D9 left radiculopial D7	Shunt and epidural venous pouch D9	No	Intradural vein	Through right L1 radiculomedullary artery with Histoacryl	Partial occlusion, without neurological changes	3
Case 3	51/F	Acute myelopathy, paraparesis (1/5 right leg; 2/5 left leg), sphincter incontinence	4	AVF extradural epidural dorsal sub type A (Rangel-Castilla). Unexpected diagnosis during spine surgery	Right D9 radiculomeningeal and radiculopial artery	Shunt and epidural venous pouch D9	No	Intradural vein	Through right D9 radiculopial artery with Histoacryl	Total occlusion, without neurological changes	4
Case 4	3/F	Chronic myelopathy (paraparesis 4/5) and lumbar pain	3	AVF pial ventral at conus medullaris	Right D11 and L2 radiculomedullary arteries	Intradural fistulous ostium at D12–L1 with venous aneurism (15 mm × 20 mm)	Venous aneurysm	L3 radiculomedullary vein	Embolized with coils and Histoacryl	Complete occlusion. Almost without sequel, paraparesis (4+/5)	1
Case 5	6/M	Chronic myelopathy (paraparesis 4/5), history of AVF embolization 5 years ago	2	AVF type IV (Di Chiro)/intradural ventral subtype A (Anson-Spetzler)	Right D9 and left D10 radiculomedullary arteries	Small ostium at D11–D12	Venous aneurysm	Venous drainage runs to anterior spinal vein	Embolized with Histoacryl	There is no evidence of AVF, same neurological status	2
Case 6	28/M	Chronic myelopathy, paraparesis (2/5 right leg; 4/5 left leg)	3	AVF type IV (Di Chiro)/intradural ventral subtype A (Anson-Spetzler) associated with tumor	Left D12 and D9 radiculomedullary arteries	Small ostium at D11	2 arterial aneurysms	Anterior spinal vein/filum terminal vein	Spontaneous thrombosis	Spontaneous thrombosis without neurological changes	3
Case 7	15/F	SAH Fisher IV, headache, neck stiffness	3	Type V dorsal CCJ AVF (Hiramatsu)	Posterior spinal artery	Middle ostium at C3	Venous aneurysm	Venous drainage to intradural varicose veins–inferior petrous vein	Embolized with Histoacryl	Complete occlusion, without neurological deficit	0
Case 8	26/F	2 episodes of syncope, chronic headache	1	Type V perimedullary CCJ AVF (Hiramatsu)/epidural dorsal (Geibprasert)	Posterior meningeal arteries, PSA, and radiculomeningeal branch from V3 on the left side	2 small ostia at foramen magnum	2 arterial aneurysm	Venous drainage to marginal sinus–epidural plexus	Embolized with Histoacryl in 2 sessions	Almost complete occlusion, without neurological deficit	0
Case 9	51/F	Chronic myelopathy, spastic paraparesis 2/5	4	Type II (Di Chiro)/compact spinal AVM (Spetzler)	Right and left D10 radiculomedullary arteries, left D9 radiculomedullary artery	Compact nidus at D10	No	Left D9 radiculomedullary vein and anterior spinal vein	Partially embolized with Histoacryl through D9 radiculomedullary artery, a second unsuccessful embolization attempt	Partial occlusion, without neurological changes	4
Case 10	18/M	Lumbar pain, acute myelopathy, paraparesis (2/5), headache, vomiting, and SAH at cranial base and perimedullary	4	Conus medullaris compact AVM (Spetzler)	Radiculomedullary arteries at left D11 and L1	Compact nidus from D12 to L2	No	Toward varicose perimedullary vein–lumbar segmentary vein	Embolized with Histoacryl in 2 sessions	Complete occlusion without sequel	0
Case 11	5/M	Acute myelopathy, hematomyelia, paraplegia, sphincter incontinence, and lumbar pain	4	Conus medullaris diffuse AVM	Right D10 and L2 RMA, left L3 RMA, right L4 RPA	Diffuse nidus with various high-flow fistulas	Arterial aneurysm at right L2 and venous aneurysm at left L2	Venous drainage runs to radiculomedullary veins and filum terminale vein	Embolized with Histoacryl (2 sessions), laminoplasty with partial resection of AVM, and hematoma evacuation	There is no evidence of AVM, clinical improvement, still with paraparesis (4/5)	2
Case 12	74/F	Chronic myelopathy, paraparesis (2/5 right leg; 4/5 left leg), sensitive level at D8, sphincter incontinence	3	Conus medullaris diffuse AVM	D7 radiculomedullary	Nidus from D11 to L1	No	Filum terminal vein and venous reflux to anterior spinal vein	Through right D7 radiculomedullary artery with Histoacryl	Total occlusion, without neurological changes	3

**Table 3:** Endovascular outcome.

Sessions of Embolization	Total Occlusion	Partial Occlusion	Total Treatments
	*n* (%)	*n* (%)	*n* (%)
1 Session	5 (50)	1 (10)	6 (60)
2 Sessions	2 (20)	2 (20)	4 (40)
Total	7 (70)	3 (30)	10 (100)

**Table 4:** Spinal cord vascular shunt classifications.

Type of Lesion	Arteriovenous Fistulas			Arteriovenous Malformations		
Subtypes by Spetzler	Extradural Epidural AVM	Intradural Dorsal AVM	Intradural Ventral AVM	Extradural Intradural	Intramedullary	Medullary Conus AVM
Pathogeny	Radicular artery to epidural venous plexus	Radicular artery to radicular or medullary vein	Anterior spinal artery to radicular or medullary vein	Metameric effect on skin, bone, muscle, and nerve tissue	1 or multiple feeders from anterior or posterior spinal arteries	1 or multiple feeders from anterior or posterior spinal arteries, 1 or multiple nidi around conus
Pathophysiology	Venous hypertension (A subtype), compression (A subtype), vascular steal, B subtype is associated with Von Recklinghausen disease	Venous congestion, rare hemorrhage	Compression (venous aneurysm), hemorrhage and vascular steal, arterial aneurysms (10%)	Compression, hemorrhage, and vascular steal	Compression, 50% debut with hemorrhage (glomus 4% to 10%), and vascular steal	Venous hypertension, compression, hemorrhage
Di Chiro classification	Without definition	Type I (dural fistula)	Type IV (Djindjian and Rosemblum)	Type III	Type II	Without definition
Subclassifications and other characteristics	By Rangel-Castilla: A: epidural drainage and perimedullary B: to Batson venous plexus B1 with compression or myelopathy B2 without compression or myelopathy	By Spetzler: A: 1 feeder B: multiple feeders By Adeeb: IA: dorsal venous plexus IB: ventral venous plexus	By Mourier and Anson-Spetzler: I/A: 1 small feeder II/B: medium size main feeder and others small feeders III/C: multipedicle lesion with great venous ectasia Associated with no metameric genetic diseases	Cobb syndrome	Compact nidus Diffuse nidus Associated with no metameric genetic diseases	Compact nidus. Glomus-like, pial perimedullary, complex venous drainage, associated with tethered cord
Subtypes by Takai	Type V (extradural) A: intradural venous drainage B: without intradural venous drainage	Type I (dural)	Type IV (perimedullary) Subtypes A, B, and C by Mourier	Type III (juvenile intramedullary)	Type II (glomus intramedullary)	Without definition

**Table 5:** Cranio-cervical junction AVF by Geibprasert.

	Ventral Epidural	Dorsal Epidural	Lateral Epidural
Venous embryology	Osteo cartilaginous (notochord) Ventral osseous drainage	Osteo membranous Osseous dorsal and leptomeningeal drainage	Leptomeningeal drainage (not related to nerves)
Localization of the shunt	Vertebral body, basioccipital sinus, sigmoid sinus, petrous pyramid, basi-sphenoidal sinus, cavernous sinus, and sphenoidal wing	Dorsal epidural spinal, dorsal part of the marginal sinus, occipital sinus, torcula, transverse sinus, superior sagittal sinus	Lateral dural spinal, lateral part of marginal sinus with emissary condylar vein, vein of Galen, basitentorial sinus, sphenoparietal sinus, paracavernous region, intraorbital and cribriform lamina
Clinics and behavior	Female (2:1), rare cortical reflux unless there is thrombosis distal to the shunt	Pediatrics, epidural hematoma, cortical reflux could occur if there is a high flow shunt or restriction of efferents	Male (4:1), elderly, aggressive behavior. Always perimedullary or cortical reflux

**Table 6:** Cranio-spinal junction AVF by Hiramatsu.

by Hiramatsu	Type I	Type II	Type III	Type IV	Type V
Denomination	Dural fistula	Radicular fistula	Epidural with pial afferents	Epidural	Perimedullary
Angioarchitecture	Meningeal afferent to intradural veinsAnd dura mater shunt	Radicular or meningeal afferents with drainage to radicular veins, Radicular shunt	Radicular or meningeal afferent with pial afferents also and epidural drainage. Shunt outside the dura mater	Radicular or meningeal afferent and epidural drainage Shunt outside the dura mater	Pial afferents with drainage to intradural veins Intradural shunt
